# RECIST Applied to Realistic Tumor Models

**DOI:** 10.6028/jres.116.013

**Published:** 2011-06-01

**Authors:** Zachary H. Levine, Benjamin R. Galloway, Adele P. Peskin

**Affiliations:** Optical Technology Division, National Institute of Standards and Technology, Gaithersburg, MD 20899-8441; Optical Technology Division, National Institute of Standards and Technology, Gaithersburg, MD 20899-8441; Department of Engineering Physics, Colorado School of Mines, Golden, CO 80401-1887; Applied and Computational Mathematics Division, National Institute of Standards and Technology, Boulder, CO 80305-3328

**Keywords:** RECIST, tumor size, volumetric measurement, x-ray imaging

## Abstract

RECIST (Response Evaluation Criteria in Solid Tumors) is a linear measure intended to predict tumor size in medical computed tomography (CT). In this work, using purely geometrical considerations, we estimate how well RECIST can predict the volume of randomly-oriented tumor models, each composed of the union of ellipsoids. The principal conclusion is that RECIST is likely to work less well for realistic tumors than for ellipsoids.

## 1. Introduction

The Response Evaluation Criteria in Solid Tumors (RECIST) [1] is used to determine whether medically significant changes have taken place in potentially cancerous lesions as imaged using computed tomography (CT). The main feature of RECIST is that the size of lesions is based on a one-dimensional measurement within planes transverse to the axis of data acquisition. The system harkens back to the display of CT images on film which was used in the late twentieth century. The lesions are three dimensional objects and ideally would be sized as such. Here, we explore computationally the measurement errors that are induced by RECIST.

## 2. RECIST With Tumor Models Based On Ellipsoids

In previous studies, we considered the measurement errors in RECIST based on measurements of physical ellipsoids [2] and randomly-oriented single ellipsoids treated theoretically [3]. Here, we study 16 model tumors which were constructed to simulate lung tumors to provide reference data as part of a larger test of volumetric measurement methods [4]. Each of the tumors was modeled with a set of 4 to 13 ellipsoids. Of these, two were nearly convex, one model was a pair of nearby tumors, and the balance showed substantial deviation from being convex. We rotate these tumors into a uniformly chosen random orientation and then we find the largest diameters in the cut plane. Our virtual measurements are performed on the geometric objects; we do not represent the objects as a series of CT slices.

The operation is somewhat more time-consuming than for the general ellipsoids [2], in that it is necessary to scan in a direction normal to the measurement plane to obtain a maximum, whereas for the ellipsoids the plane containing the origin would contain the RECIST diameter. An additional complication occurs because the tumor models are not necessarily convex. Hence, the possibility of having more than one isolated two-dimensional region in the cut plane appears. We decided to keep the largest two such values, which is in keeping with the rule of RECIST 1.1 that up to two tumors per organ may be studied [1]. Although these disjoint regions may belong to the same tumor, we are assuming that our “radiologist” would not consider a connection using information from other CT slices and interpreted the regions as being two tumors [5].

**Fig. 1:**
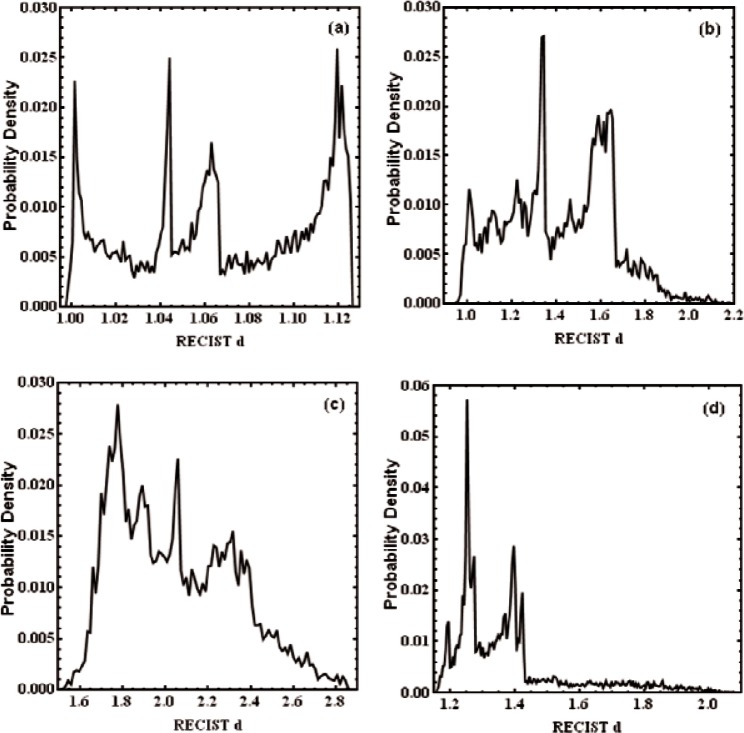
Probability densities of four sampled tumor models with random orientations and normalized volume *V* = π / 6. The tumors chosen had probability densities with (a) the smallest $\sigma_{d}/\bar{d}$, (b) the largest $\sigma_{d}/\bar{d}$, (c) the largest $\bar{d}$, and (d) the largest values for both skewness and kurtosis.

We normalize the volumes to π/6 so that the RECIST diameter *d* = 1 would be produced for spherical objects. We present the distributions of RECIST values for four model tumors in Fig. 1 which represent the extremes of the 16 distributions. The mean of a given distribution is denoted by $\bar{d}$ and its standard deviation by *σ_d_*. The model with the smallest ratio of $\sigma_{d}/\bar{d}$, which is roughly spherical with two pairs of lobes, has peaks at the extremes of Fig. 1a which resemble peaks predicted for the uniaxial distribution in Fig. 1 of Ref. [3]. The distribution in Fig. 1b is notable for a long, low tail which arises when the object appears in two parts in a cut plane. A similar figure is shown in Fig. 1d. These figures are remarkable for their structure: individual tumor models give rise to highly structured RECIST value distributions, but these distributions do not resemble each other. The distribution with the largest $\bar{d}$ value is shown in Fig. 1c; this model was the pair of closely positioned tumors.

**Fig. 2:**
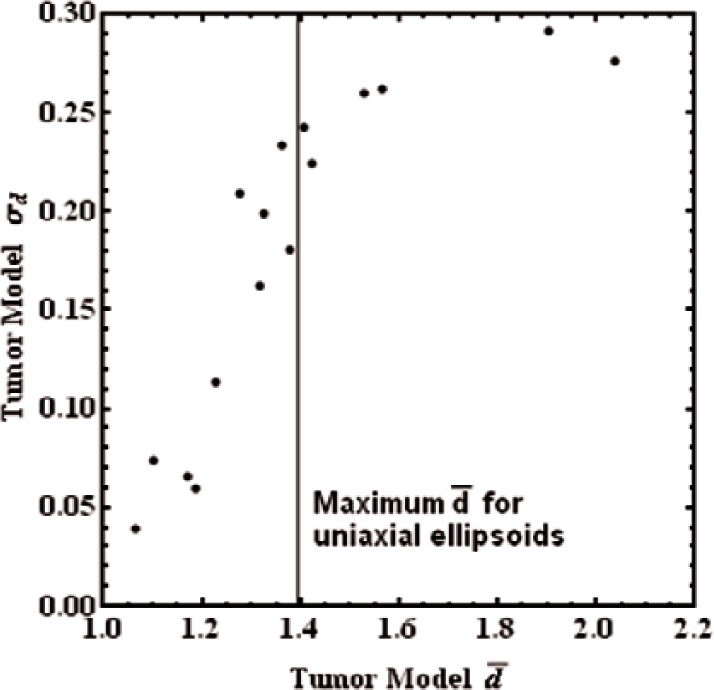
Standard deviation of the RECIST diameter distributions for each of the 16 tumor models as a function of their average diameters. The vertical line shows the maximum RECIST diameter for uniaxial ellipsoids according to Fig. 2a of Ref. [3].

In Fig. 2, we present the standard deviation of the RECIST value as a function of the mean RECIST value. (Recall all volumes are normalized to π/6 which yields *d* = 1 for a sphere.) The uniaxial ellipsoid limit, i.e., the maximum orientationally-averaged RECIST value for a uniaxial ellipsoid with any ratio of its axes, [3] is shown in the figure. Six of sixteen model tumors exceed this value. The standard deviations are correlated with the mean diameter value. That is, tumors with irregular shapes produce large values, but they do so in a way which is hard to predict in individual cases.

**Fig. 3:**
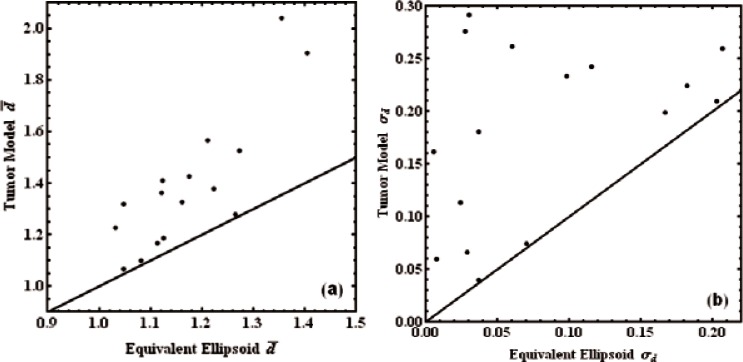
(a) Mean RECIST diameters for each of the 16 tumor models compared to the mean RECIST diameters for ellipsoids with equal second moments. (b) Same comparison for standard deviations. The 1:1 lines are shown.

Finally, in Fig. 3, we compare the mean RECIST values and standard deviations of the 16 realistic tumors to those of particular, randomly oriented, general ellipsoids. The three parameters *a*, *b*, and *c* for each of the ellipsoids were chosen to match the eigenvalues of the second moment tensors of the tumor models. All 16 model tumor values lie above the 1:1 lines, indicating that the ellipsoid model probably overestimates the ability of RECIST to predict tumor volumes.

## 3. Discussion and Conclusions

Our studies of more realistic tumor models suggest that the randomly-oriented ellipsoid model underestimates the uncertainty of RECIST in predicting tumor volumes. Werner-Wasik et al. [6] and Rossi et al. [7] describe tumor volumes as irregular. Li et al. [8] find that among nodules in the lung, malignant ones tend to have a round or complex shape, whereas benign lesions have these shapes as well as oval and polygonal shapes. Takashima et al. [9] report that malignancies are more spherical than benign lesions for solitary pulmonary nodules no larger than 1 cm. If the tumors have a complex shape, our results on the more realistic tumor models show that additional uncertainty is very likely. More subtly, if the malignancies are more spherical than benign lesions, RECIST will preferentially select benign lesions for study. In practice, sometimes highly complicated lesions are deemed “unmeasureable” and are excluded from further study [1], leading to a different kind of selection bias.

The general conclusion of this work is that the measurement errors induced by RECIST compared to volume measurements for single ellipsoids studied previously [3] is very likely to be a lower bound on the measurement errors in real tumors.
